# A Single-Domain Antibody-Based Anti-PSMA Recombinant Immunotoxin Exhibits Specificity and Efficacy for Prostate Cancer Therapy

**DOI:** 10.3390/ijms22115501

**Published:** 2021-05-23

**Authors:** Yutong Xing, Keyuan Xu, Shixiong Li, Li Cao, Yue Nan, Qiyu Li, Wenjing Li, Zhangyong Hong

**Affiliations:** State Key Laboratory of Medicinal Chemical Biology, College of Life Sciences, Nankai University, Tianjin 300071, China; xingyutong@mail.nankai.edu.cn (Y.X.); 1120190518@mail.nankai.edu.cn (K.X.); 2120191089@mail.nankai.edu.cn (S.L.); 1120150370@mail.nankai.edu.cn (L.C.); 2120201117@mail.nankai.edu.cn (Y.N.); qiyuli@mail.nankai.edu.cn (Q.L.); 1120160385@mail.nankai.edu.cn (W.L.)

**Keywords:** prostate carcinoma, immunotoxins, sdAbs (single-domain antibodies), PE, PSMA (prostate-specific membrane antigen)

## Abstract

Prostate cancer (PCa) is the second most common cancer in men, causing more than 300,000 deaths every year worldwide. Due to their superior cell-killing ability and the relative simplicity of their preparation, immunotoxin molecules have great potential in the clinical treatment of cancer, and several such molecules have been approved for clinical application. In this study, we adopted a relatively simple strategy based on a single-domain antibody (sdAb) and an improved *Pseudomonas* exotoxin A (PE) toxin (PE24X7) to prepare a safer immunotoxin against prostate-specific membrane antigen (PSMA) for PCa treatment. The designed anti-PSMA immunotoxin, JVM-PE24X7, was conveniently prepared in its soluble form in an *Escherichia coli* (*E. coli*) system, avoiding the complex renaturation process needed for immunotoxin preparation by the conventional strategy. The product was very stable and showed a very strong ability to bind the PSMA receptor. Cytotoxicity assays showed that this molecule at a very low concentration could kill PSMA-positive PCa cells, with an EC_50_ value (concentration at which the cell viability decreased by 50%) of 15.3 pM against PSMA-positive LNCaP cells. Moreover, this molecule showed very good killing selectivity between PSMA-positive and PSMA-negative cells, with a selection ratio of more than 300-fold. Animal studies showed that this molecule at a very low dosage (5 × 0.5 mg/kg once every three days) completely inhibited the growth of PCa tumors, and the maximum tolerable dose (MTD) was more than 15 mg/kg, indicating its very potent tumor-treatment ability and a wide therapeutic window. Use of the new PE toxin, PE24X7, as the effector moiety significantly reduced off-target toxicity and improved the therapeutic window of the immunotoxin. The above results demonstrate that the designed anti-PSMA immunotoxin, JVM-PE24X7, has good application value for the treatment of PCa.

## 1. Introduction

Prostate cancer (PCa), a prostatic epithelial malignant tumor, is the second most common cancer in men, accounting for more than one-fifth of newly diagnosed cancer cases in this population and causing more than 300,000 deaths every year worldwide [1]. Whereas primary PCa tumors can be managed quite successfully, there are no curative treatments for those in advanced stages, with 18% of PCa patients dying of this disease each year [2]. Among patients with advanced PCa, a large proportion will develop hormone-independent or hormone-resistant PCa, and effective drugs and treatment methods are lacking, giving only a 15% 5-year survival rate. Once PCa enters the middle and advanced stages, it shows a high rate of cancer metastasis, which increases the difficulty of treatment and seriously reduces patient quality of life. It is therefore of great significance to identify more effective drugs and treatment regimens for the clinical therapy of PCa [3,4,5].

Prostate-specific membrane antigen (PSMA), also known as glutamic acid carboxypeptidase II, is a type II transmembrane glycoprotein and an ideal target for the treatment of PCa [6,7,8]. It is highly expressed in almost all PCas, including metastatic PCas and internalized after ligand binding; however, PSMA is not expressed at all or expressed at only extremely low levels in normal tissues. Some targeted drug molecules against PSMA show good application potential against PCa [9,10,11]. For example, the monoclonal antibody J591 entered phase II clinical trials and caused prostate-specific antigen (PSA) to be stable in some patients when combined with low-dose interleukin-2, although the lethal effects of J591 alone against tumors were not very strong [12]. The antibody drug conjugates (ADCs) MLN2704 [13] and PSMA-ADC [14] entered phase II clinical trials and showed successful therapeutic effects, although no follow-up studies have been carried out, which may be due to the deconjugatation of small-molecule toxins, which results in peripheral neuropathy and limits the therapeutic window. Additionally, the small-molecule ligand radionuclide-coupled diagnostic agent ^68^Ga-PSMA-11 was approved in 2020 by the U.S. Food and Drug Administration (FDA) for the clinical diagnosis of PCa [15]. These studies suggest that PSMA is an ideal target for the treatment of PCa.

Recombinant immunotoxins (RITs) are a class of antibody-fusing drug molecules that fuse protein toxin molecules to significantly increase the killing ability of antibody molecules against cancer cells. At present, the following three immunotoxin molecules have been approved by the FDA for the clinical treatment of tumors: denileukin diftitox (Ontak; Eisai Inc., Woodcliff Lake, NJ, USA, approved in 1999) [16], which fuses human interleukin-2 with truncated diphtheria toxin; moxetumomab pasudotox (Lumoxiti; AstraZeneca, approved in 2018) [17], which fuses an anti-CD22 antibody fragment with a 38-kD *Pseudomonas* exotoxin A (PE) toxin fragment (PE38); and tagraxofusp-erzs (Elzonris; Stemline Therapeutics, approved in 2018) [18], which fuses human interleukin-3 to truncated diphtheria toxin (DAB389). Toxin proteins are extremely cytotoxic as the entrance of one or several molecules into the cytoplasm is enough to kill the cell; their effects are substantially stronger than those of the microtubule inhibitors and DNA damage drugs commonly used for the construction of ADCs [19,20,21,22]. Moreover, the preparation of immunotoxin molecules is relatively simple, and their molecular structure is completely homogeneous [23,24], which can avoid the complex preparation processes of ADC drug molecules and side effects caused by the early release of small-molecule toxins from ADC drug molecules. These factors make immunotoxins intriguing to a broad audience. At present, some studies have attempted to develop such molecules for the treatment of PCa, such as the immunotoxin A5-PE40, which showed therapeutic efficacy in animal tumor models [25], and the development of immunotoxins targeting PSMA is a new strategy to treat PCa.

However, there are some limitations to the clinical application of immunotoxins prepared according to the conventional strategy in which a single-chain variable fragment of an antibody (scFv) or its disulfide-stabilized derivative (dsFv) is fused with the PE38 toxin. These molecules have relatively high off-target toxicities and immunogenicity, and their relatively large molecular weights may reduce their ability to permeate solid tumors. In addition, due to the ease in preparing scFvs or dsFvs in *Escherichia coli* (*E. coli*) systems and the toxicity of the toxin proteins to other expression systems, the preparation of such molecules is complicated by expression in the form of inclusion bodies in the *E. coli* system, which is followed by a complex renaturation process [26,27]. In view of the above problems, we intended to adopt a more convenient strategy to prepare immunotoxins targeting PSMA based on a single-domain antibody (sdAb) and an improved PE toxin.

Unlike scFvs or dsFvs, sdAbs, also known as nanobodies or VHH molecules, contain only a heavy chain variable region [28], so they are substantially smaller (approximately 120 amino acids; only half that of the former) and, importantly, they can be well expressed in their soluble form in a bacterial system [29,30,31]. In addition, sdAbs have ideal clinical safety; currently, the sdAb-based drug caplacizumab (Cablivi; Sanofi; approved in 2018 by the FDA) is used in the clinical treatment of acquired thrombocytopenic purpura (attP) [32,33]. PE24X7 is an improved version of a PE toxin protein with seven point mutations in domain III and the deletion of most of domain II, unlike the conventional PE38 toxin. Previous studies have shown that the PE24X7 toxin has relatively lower immunogenicity and off-target toxicity but still has almost the same cytotoxicity as the PE38 toxin [34]. Therefore, the design of immunotoxins against PSMA based on sdAbs and the improved PE24X7 toxin may have better clinical application potential. In this study, we designed such a novel immunotoxin (JVM-PE24X7) and tested its potential in the treatment of PCa through detailed biochemical, cellular and animal experiments.

## 2. Results and Discussion

### 2.1. Expression and Characterization of the Immunotoxin JVM-PE24X7

**Figure 1:**
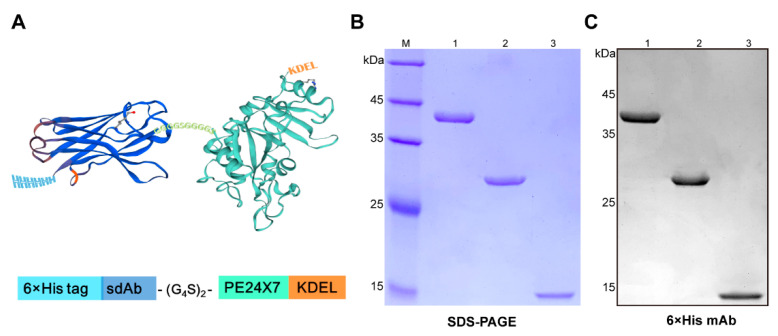
Characterization of the immunotoxin JVM-PE24X7. (**A**) An illustrated structure of JVM-PE24X7 constructed by fusing the sdAb (dark blue) with the toxin PE24X7 (mint green). (**B**) SDS-PAGE and (**C**) Western blot analysis of purified JVM-PE24X7 (lane 1), PE24X7 (lane 2) and JVM (lane 3). The proteins were loaded onto a 12.5% polyacrylamide gel with protein weight standards and detected by Coomassie brilliant blue staining or Western blot analysis using a mouse anti-His monoclonal antibody.

Construction of the expression plasmid for the anti-PSMA immunotoxin JVM-PE24X7 was based on the sequences of the anti-PSMA sdAb JVM and the optimized PE toxin PE24X7 (Figure 1A). The anti-PSMA sdAb JVM was reported to have ideal binding strength and selectivity for the PSMA receptor and can be expressed in its soluble form in high yield in an *E. coli* system. The optimized toxin PE24X7 lacked most of the sequence of domain II of the commonly used PE38 toxin, which was suggested to lead to lower immunogenicity. At the same time, seven sites in domain III were mutated to further reduce the immunogenicity of the toxin, and the C-terminal sequence of PE24X7 was changed to KDEL to increase the efficiency of toxin escape from lysosomes. The two sequences were linked by a flexible (G_4_S)_2_ linker (G_4_S, Gly-Gly-Gly-Gly-Ser), and a His-tag was added at the N-terminus to facilitate purification. The linked sequence was inserted into the pET-22b (+) vector between the NcoI and XhoI restriction digestion sites. In addition, the JVM sequence and PE24X7 sequence were similarly inserted into the pET-22b (+) expression vector to construct plasmids for expression of the sdAb JVM protein and free PE24X7 toxin protein as controls.

The plasmids were transformed into *E. coli* BL21 (DE3) cells for expression, followed by purification with nickel affinity chromatography. The purity of the proteins was determined by 12.5% sodium dodecyl sulfate-polyacrylamide gel electrophoresis (SDS-PAGE), and all showed a purity greater than 95% with a molecular size consistent with that obtained by theoretical calculation (Figure 1B). Western blot analysis based on an anti-His tag antibody further verified the purities and molecular sizes of the proteins (Figure 1C). The storage stability of JVM-PE24X7 was evaluated by detecting the relative activity of the immunotoxin under different storage conditions. As shown in Figure S1, JVM-PE24X7 was very stable after 7 days of storage at 4 °C and 2 months of storage at −20 °C and −80 °C, retaining more than 95% activity. Matrix-assisted laser desorption ionization-time of flight (MALDI-TOF) mass spectrometry (Figure S2) corroborated the identities of JVM-PE24X7 (38193.2 Da; theoretical molecular mass, 38172.4 Da) and JVM (14677.4 Da; theoretical molecular mass, 14621.4 Da).

### 2.2. Expression Levels of PSMA Receptors in PCa Cells

**Figure 2:**
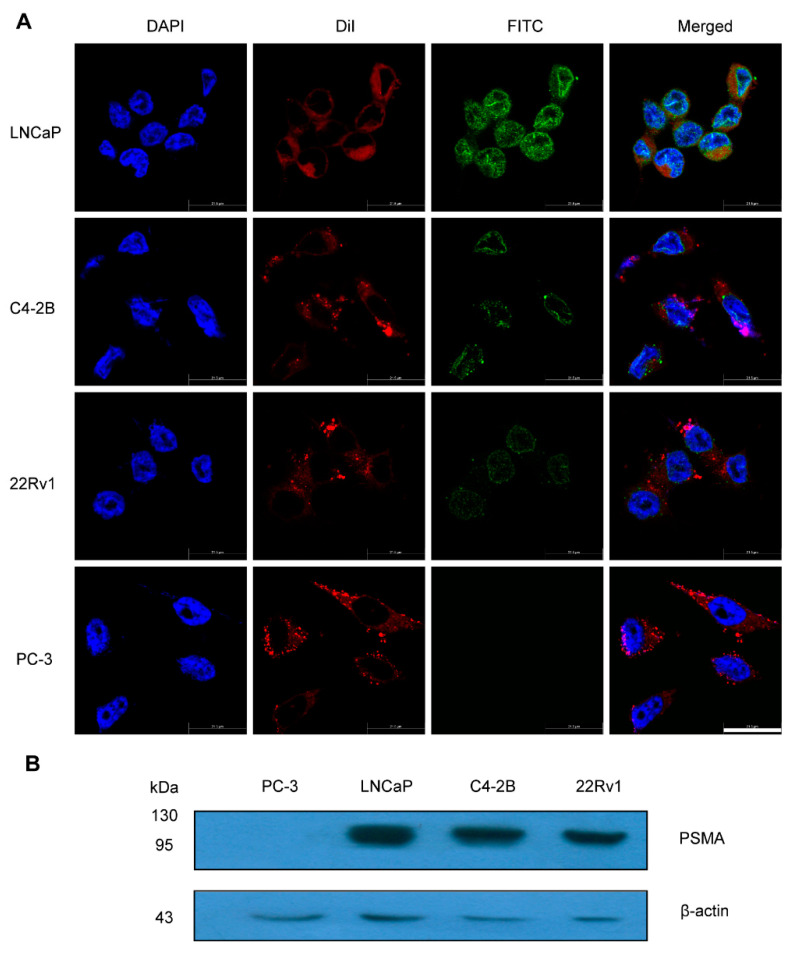
Expression levels of PSMA in a variety of PCa cells. (**A**) LNCaP, C4-2B, 22Rv1 and PC-3 cells were fixed on slides and sequentially incubated with an anti-PSMA antibody and a FITC-labeled secondary antibody (green) DiI was used for staining of cell membrane (red). DAPI is used for nuclear staining (blue) (magnification 2400×). Scale bar: 21.5 μm. (**B**) Whole-cell lysates from LNCaP, C4-2B, 22Rv1 and PC-3 cells were analyzed by Western blotting.

The immunotoxin JVM-PE24X7 binds to the PSMA receptor, through which it enters the cytoplasm via receptor-mediated endocytosis and glycosylates ribosomes to inhibit protein synthesis, thus killing the cell. Therefore, the expression level of the PSMA receptor on the cell surface is very important for the antitumor function of JVM-PE24X7. We detected PSMA expression in LNCaP, C4-2B, 22Rv1 and PC-3 cells by immunofluorescence staining and Western blot analysis. According to the immunofluorescence analysis in Figure 2A and Western blot analysis in Figure 2B, PSMA was highly expressed in LNCaP, C4-2B and 22Rv1 cells but showed very low expression in PC-3 cells. Therefore, LNCaP, C4-2B and 22Rv1 cells were used as PSMA-positive cells, while PC-3 cells were used as PSMA-negative cells to evaluate the biological activity of the immunotoxin JVM-PE24X7. Notably, LNCaP cells expressed more PSMA than the other two PSMA-positive cell lines.

### 2.3. Binding Affinity Analysis of JVM-PE24X7 towards PSMA Receptors Determined by ELISA

**Figure 3:**
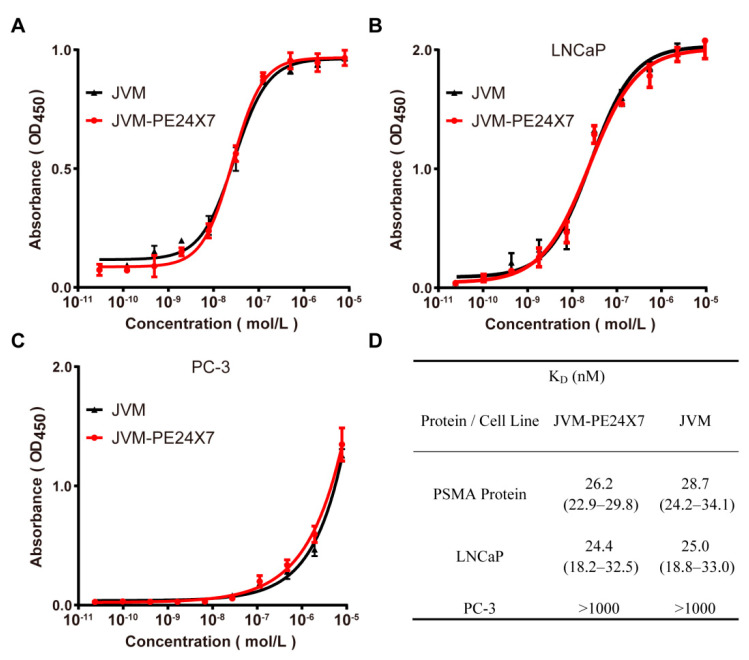
JVM-PE24X7 binding assays towards the PSMA receptor by ELISA. (**A**) Protein-based ELISA in which the human PSMA protein was coated on plates to determine the binding affinity of JVM-PE24X7 for the PSMA protein. (**B**&**C**) Cell-based ELISA in which PSMA-positive LNCaP cells or PSMA-negative PC3 cells were seeded onto the plate to determine the binding of JVM-PE24X7 to the PSMA receptor in the natural environment. (**D**) The K_D_ data were calculated using GraphPad Prism.

We measured the binding affinity of the immunotoxin JVM-PE24X7 to its corresponding receptor, PSMA, by enzyme-linked immunosorbent assay (ELISA). First, protein-based ELISA was adopted for this measurement by plating the human PSMA protein on a plate and incubating it with JVM-PE24X7, followed by ELISA detection. Here, the anti-PSMA sdAb JVM was used as a control. As shown in Figure 3A, JVM-PE24X7 and JVM showed a high binding affinity for the PSMA protein; on the other hand, there was no difference in binding ability with the human PSMA protein between JVM-PE24X7 and JVM, which indicated that fusion with PE24X7 did not significantly affect the ability of the sdAb JVM to bind the PSMA receptor. PE24X7 was fused at the C-terminus of JVM, which is distant from the binding region between JVM and its receptor. In addition, the flexible linker (G_4_S)_2_ may have also helped to reduce the effects of fusion on the antibody structure.

To more realistically simulate the binding of JVM-PE24X7 to its receptors, cell-based ELISA was used to evaluate the binding affinity of JVM-PE24X7 for its receptors on the cell surface. Here, PSMA-positive LNCaP cells and PSMA-negative PC-3 cells were adopted for the experiment. As shown in Figure 3B (LNCaP cells) and Figure 3C (PC-3 cells), JVM-PE24X7 and JVM showed a high binding affinity for LNCaP cells but almost no affinity for PC-3 cells, showing good dependence on receptor expression levels.

Figure 3D shows the calculated equilibrium dissociation constant (K_D_) determined by GraphPad Prism (version X; La Jolla, CA, USA). In the protein-based ELISA, JVM-PE24X7 and JVM showed K_D_ values against the PSMA protein of 26.2 nM and 28.7 nM, respectively. In the cell-based ELISA, JVM-PE24X7 and JVM showed K_D_ values against PSMA-positive LNCaP cells of 24.4 nM and 25.0 nM, respectively, while they both showed substantially increased K_D_ values (higher than 1000 nM) against PSMA-negative PC-3 cells. These data are consistent and indicate that JVM-PE24X7 has an ideal high-intensity binding affinity and selectivity for its target receptor.

### 2.4. Cellular Internalization of JVM-PE24X7 into LNCaP and PC-3 Cells

**Figure 4:**
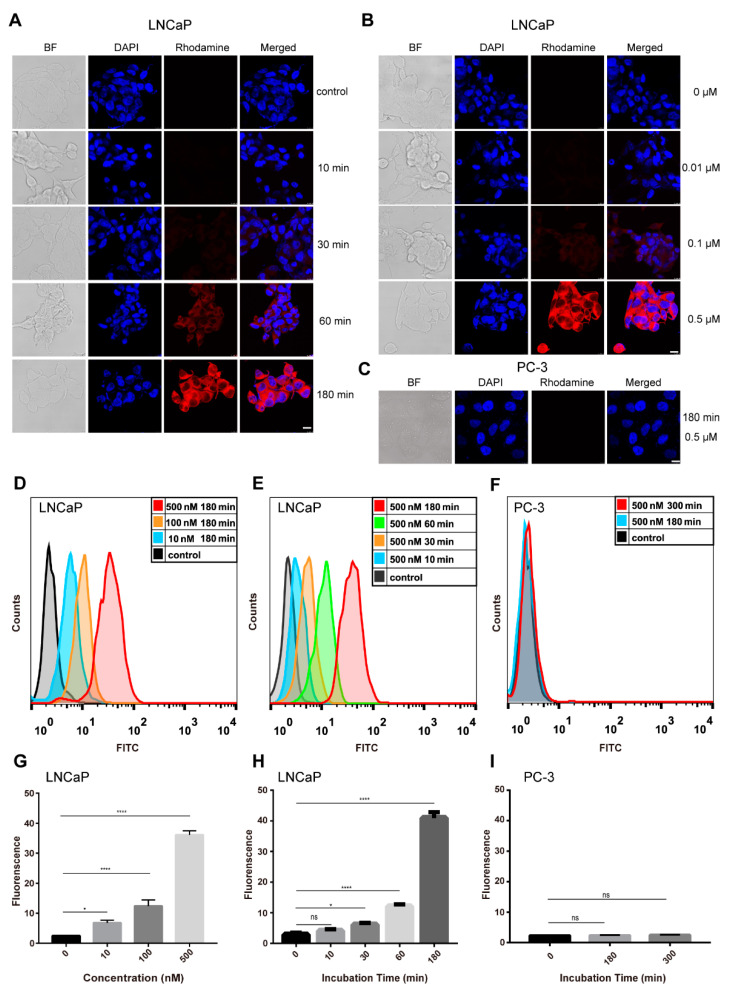
Fluorescence microscopy and flow cytometry analyses of JVM-PE24X7 internalization and accumulation in LNCaP and PC-3 cells. (**A**) LNCaP cells were treated with 0.5 μM rhodamine-labeled JVM-PE24X7 for 10 min, 30 min, 60 min, or 180 min. (**B**) LNCaP cells were treated with 0, 0.01, 0.1 or 0.5 μM rhodamine-labeled JVM-PE24X7 for 180 min. (**C**) PC-3 cells were treated with 0.5 μM rhodamine-labeled JVM-PE24X7 for 180 min. After washing with phosphate-buffered saline (PBS), cells were fixed and incubated with DAPI. The cells were observed under a fluorescence microscope (magnification 400×). Scale bar: 10 μm. (**D**) LNCaP cells were treated with 0.01, 0.1, or 0.5 μM FITC-labeled JVM-PE24X7 for 180 min. (**E**) LNCaP cells were treated with 0.5 μM FITC-labeled JVM-PE24X7 for 10, 30, 60 or 180 min. (**F**) PC-3 cells were treated with 0.5 μM FITC-labeled JVM-PE24X7 for 180 or 300 min. After washing with PBS, the cells were collected and analyzed by flow cytometry. (**G**–**I**) The fluorescence data in **D**–**F** were all quantified. Error bars, means ± SEM (*n* = 3). Statistical significance was calculated by Student’s *t*-test: * *p* < 0.05; **** *p* < 0.0001. ns = no significance.

Fluorescence microscopy and flow cytometry were used to measure the cellular binding and internalization of JVM-PE24X7. Immunotoxins based on PE need to bind cells and enter the cytoplasm to exert their cytotoxic effects by inhibiting protein synthesis. Therefore, whether JVM-PE24X7 could be internalized into the cytoplasm and the efficiency of its internalization are very important for understanding the cytotoxic activity of JVM-PE24X7. Here, JVM-PE24X7 was first labeled with rhodamine B or FITC for convenient microscopic observation and flow cytometry analysis. In the microscopy experiment, PSMA-positive LNCaP cells or PSMA-negative PC-3 cells were incubated with rhodamine B-labeled JVM-PE24X7 for various duration or at various concentrations. LNCaP cells showed obviously increased fluorescence intensity with increasing incubation time (from 10 min to 180 min, Figure 4A) or rhodamine B-labeled JVM-PE24X7 concentration (from 0.01 μM to 0.5 μM, Figure 4B). In contrast, no fluorescence signal from rhodamine B was detected in PC-3 cells even after incubation with a high concentration (0.5 μM) of rhodamine B-labeled JVM-PE24X7 for a long time (180 min) (Figure 4C).

Similar results were obtained by flow cytometry analysis. As shown in Figure 4D–I, the accumulation of FITC-labeled JVM-PE24X7 in LNCaP cells gradually increased in an obvious time- and dose-dependent manner. When the cells were incubated with 0.5 μM FITC-labeled JVM-PE24X7 for 180 min, a very high concentration of immunotoxin accumulated in LNCaP cells. However, no obvious accumulation of FITC-labeled JVM-PE24X7 was detected in PC-3 cells under these conditions. The above experiments show that the synthesized immunotoxin JVM-PE24X7 can bind and be internalized by PSMA-positive cells and shows very good receptor-dependent characteristics.

### 2.5. In Vitro Cytotoxicity of Immunotoxins

**Figure 5:**
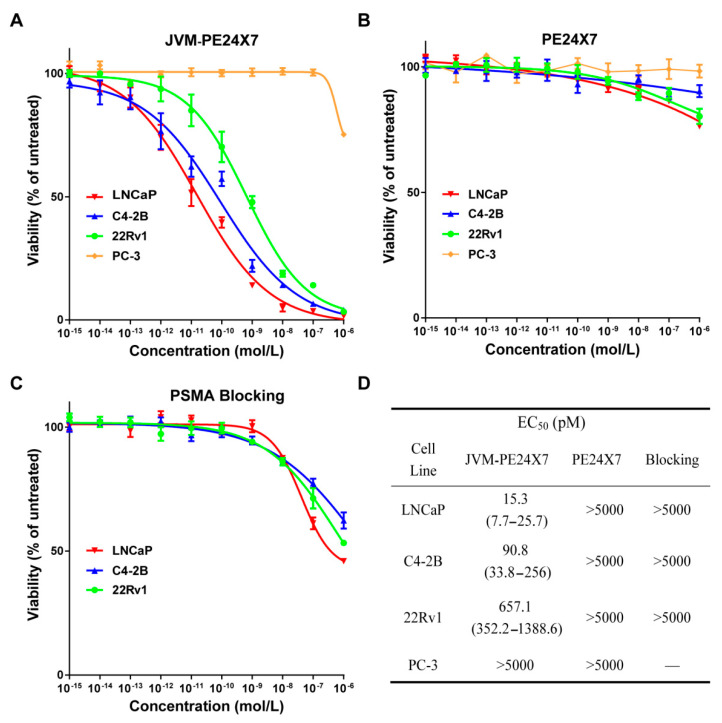
PSMA-positive LNCaP, C4-2B, and 22Rv1 cells and PSMA-negative PC-3 cells were incubated with (**A**) JVM-PE24X7 or (**B**) PE24X7 for 72 h or (**C**) preincubated with 1.0 μM JVM for 1 h before JVM-PE24X7 was added. Then, the cell viabilities were determined by MTT assay. (**D**) EC_50_ data were calculated with GraphPad Prism. Error bars, means ± SEM (*n* = 5).

An MTT assay was used to determine the cytotoxicity of JVM-PE24X7 to PSMA-positive LNCaP, C4-2B, and 22Rv1 cells and PSMA-negative PC-3 cells in vitro (Figure 5). As shown in Figure 5A, JVM-PE24X7 showed extremely strong cytotoxicity to PSMA-positive cells, with EC_50_ values (concentrations at which the cell viability decreased by 50%) as low as 15.3 pM (LNCaP cells), 90.8 pM (C4-2B cells) and 657 pM (22Rv1 cells) (Figure 5D), which are far beyond the reach of common antibodies or other protein drugs, showing the superior cytotoxic capacity of immunotoxins. In contrast, JVM-PE24X7 did not show obvious cytotoxicity towards PSMA-negative PC-3 cells. Even when the concentration was increased to 10 nM, PC-3 cell viability was still close to 100%. These data show that JVM-PE24X7 has extremely strong activity towards PSMA-positive cells and extremely high selectivity for the PSMA receptor. Compared with that towards PSMA-negative cells, the cytotoxicity of JVM-PE24X7 towards PSMA-positive cells was increased nearly 300-fold. In addition, with increasing PSMA receptor expression on 22Rv1, C4-2B and LNCaP cells, the EC_50_ values of JVM-PE24X7 towards these cells also showed clear decreases, further confirming the correlation between JVM-PE24X7 cytotoxicity and the expression level of the PSMA receptor on the cell surface. The free toxin PE24X7 was not cytotoxic towards these cells (Figure 5B,D).

In addition, a receptor-blocking experiment was adopted to further evaluate the role of the PSMA receptor in the process of JVM-PE24X7 cell killing. Here, PSMA-positive LNCaP, C4-2B and 22Rv1 cells were pretreated with 1.0 μM sdAb JVM to block PSMA receptors and then coincubated with JVM-PE24X7 for an MTT assay. As shown in Figure 5C, after blocking the receptors, the cytotoxicity of JVM-PE24X7 towards these cells was significantly reduced, and the corresponding EC_50_ value increased to 50 nM, 4.1 μM and 0.67 μM, respectively (Figure 5D). These data further confirmed that the cytotoxicity of JVM-PE24X7 towards cells is dependent on the PSMA receptor.

**Figure 6:**
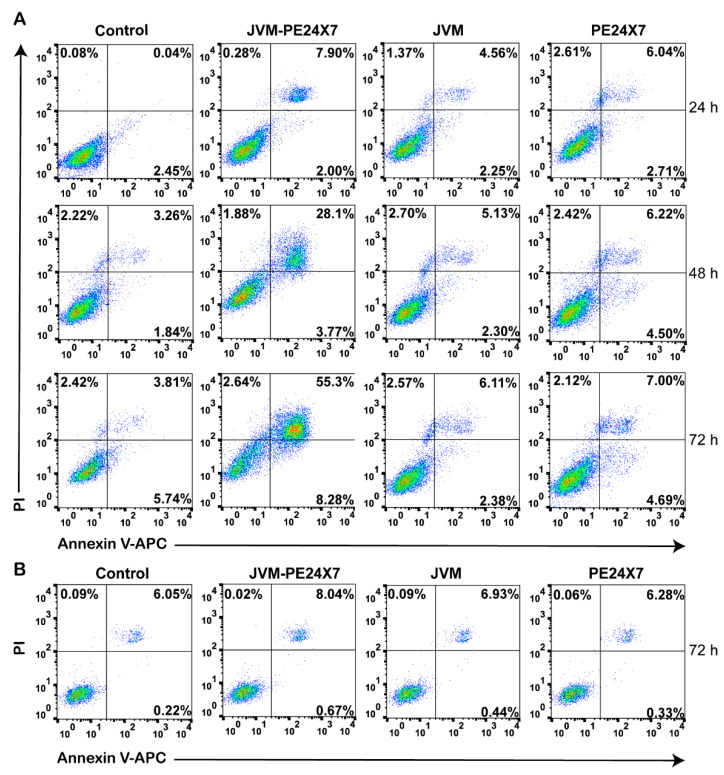
Representative flow cytometry plots of PSMA-positive C4-2B cells and PSMA-negative PC-3 cells treated with JVM-PE24X7, JVM or PE24X7. (**A**) C4-2B cells were treated with 100 pM JVM-PE24X7, JVM or PE24X7 for 24, 48 or 72 h. (**B**) PC-3 cells were treated with 100 nM JVM-PE24X7, JVM or PE24X7 for 72 h, stained with annexin V-APC/PI and analyzed for apoptosis by flow cytometry.

Annexin V-APC/PI staining was used to detect specific apoptosis induced by JVM-PE24X7. As shown in Figure 6A, when PSMA-positive C4-2B cells were treated with 100 pM JVM-PE24X7, the cells showed a significant increase in apoptosis. Moreover, with prolonged drug incubation time, the apoptosis rate significantly increased, reaching 50–60% at 72 h. In the control groups treated with phosphate-buffered saline (PBS), sdAb JVM and PE24X7, almost no apoptosis occurred, with apoptosis rates of less than 8% at 72 h, and there was no significant difference between the three control groups. When PSMA-negative PC-3 cells were incubated with JVM-PE24X7, almost no effective apoptosis was induced, with an apoptosis rate lower than 10% after 72 h of incubation with JVM-PE24X7 at a concentration as high as 100 nM (Figure 6B). Western blot anti-caspase-3 was also used to detect specific apoptosis induced by JVM-PE24X7. As shown in Figure S4, with the extension of incubation time of 100 pM JVM-PE24X7 on PSMA positive C4-2B cells, the degree of caspase-3 cleavage was gradually increased. These results indicate that the immunotoxin JVM-PE24X7 induces a significant increase in apoptosis in a PSMA receptor-dependent manner. However, apoptosis levels were low in PSMA receptor-negative cells and in molecules lacking monoclonal antibodies or part of the PE toxin.

### 2.6. In Vitro Observations by Live/Dead Staining

**Figure 7:**
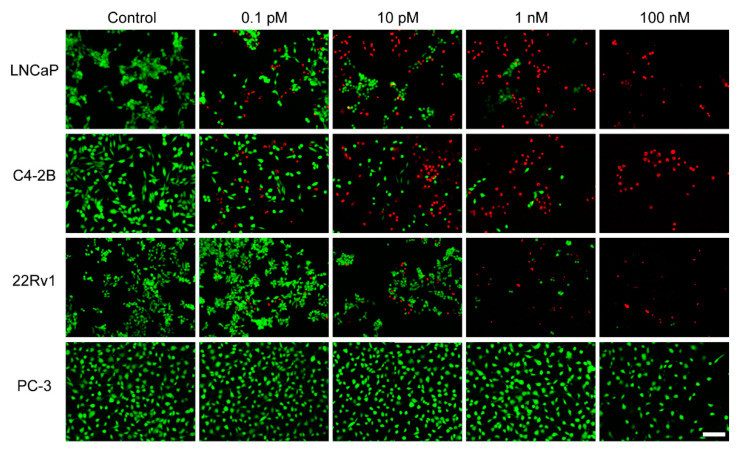
Cytotoxicity of JVM-PE24X7 visualized by using live/dead staining. PSMA-positive LNCaP, C4-2B, and 22Rv1 cells and PSMA-negative PC-3 cells were incubated with various concentrations of JVM-PE24X7 (0 M, 0.1 pM, 10 pM, 1 nM or 100 nM) and then visualized by microscopic imaging. The live and dead cells were stained with calcein-AM (green) and EthD-1 (red), respectively. Scale bar: 50 μm.

Live/dead staining was used to further visualize the cytotoxicity of JVM-PE24X7. Here, PSMA-positive LNCaP, C4-2B, and 22Rv1 cells and PSMA-negative PC-3 cells were incubated with 0.1 pM, 10 pM, 1.0 nM or 100 nM JVM-PE24X7. The living and dead cells were stained with calcein-AM (green) and EthD-1 (red), respectively, and then observed by microscopy. As shown in Figure 7, when treated with different concentrations of JVM-PE24X7, almost all PSMA-negative PC-3 cells were green, similar to those without JVM-PE24X7 treatment, which indicates that JVM-PE24X7 at these concentrations did not have an obvious killing effect on PC-3 cells. In contrast, after treatment with 10 pM JVM-PE24X7, approximately half of the PSMA-positive LNCaP and C4-2B cells and 20% of the PSMA-positive 22Rv1 cells were dead. When the concentration increased to 1.0 nM, most of these PSMA-positive cells were dead. The results of these visualization experiments are consistent with the results of MTT measurements, which further confirms the superior cytotoxicity of JVM-PE24X7 towards PSMA-positive cancer cells.

### 2.7. In Vivo Antitumor Activity of JVM-PE24X7 in a Xenograft Model

**Figure 8:**
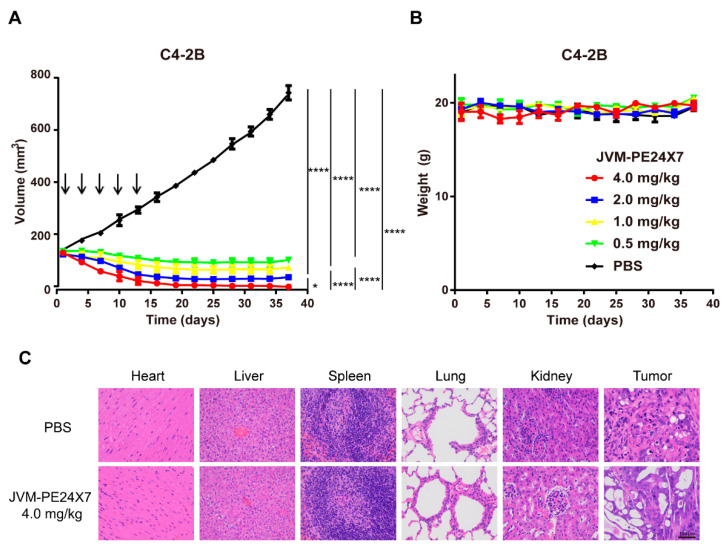
In vivo antitumor activity of JVM-PE24X7. Mice bearing C4-2B tumors were treated with the immunotoxin JVM-PE24X7 or PBS on days 1, 4, 7, 10 and 13. (**A**) Changes in tumor volume and (**B**) body weight in the mice were measured every other day. Arrows indicate the time points of JVM-PE24X7 injection. (**C**) Histological analysis of the tumor and major tissues after treatment with PBS and JVM-PE24X7 (400×). Scale bar: 100 μm. Error bars, means ± SEM (*n* = 5). Statistical significance was calculated by Student’s *t*-test: * *p* < 0.05; **** *p* < 0.0001.

A subcutaneous xenograft tumor model was used to evaluate the in vivo antitumor activity of JVM-PE24X7. Here, PSMA-positive C4-2B tumor cells (2 × 10^7^ cells per mouse) were injected subcutaneously into the right flanks of male NOD-SCID mice. After the tumor volumes reached approximately 140 mm^3^, the mice were randomized into five groups and injected intravenously with JVM-PE24X7 at five doses (0.5, 1.0, 2.0 or 4.0 mg/kg) or PBS on days 1, 4, 7, 10 and 13. The tumor volumes and body weights are shown in Figure 8. As displayed in Figure 8A, JVM-PE24X7 at different doses significantly inhibited tumor growth in the mice. When the mice were treated with a very low dose of JVM-PE24X7 (0.5 mg/kg), tumor growth was immediately inhibited, and the tumor volume was even partially reduced from approximately 160 mm^3^ at the beginning of the treatment to approximately 140 mm^3^ at the end of the monitoring period (day 37 after the first injection). When the dose was increased to 1.0 mg/kg or 2.0 mg/kg, the reduction in the tumor volume was enhanced, from an initial volume of 160 mm^3^ to approximately 80 mm^3^ and 45 mm^3^ at the end of the monitoring period (day 37), respectively. When the treatment dose was further increased to 4.0 mg/kg, the tumor shrank very rapidly. At the end of the monitoring period (day 37), only a small fibrotic lump with a volume of approximately 15 mm^3^ remained in the tumor site. In contrast, tumor progression was rapid in the control group injected with PBS, and the average tumor volume had increased to approximately 800 mm^3^ on day 37.

The above results clearly demonstrate that JVM-PE24X7 has very good tumor treatment ability. Even at a very low dose (0.5 mg/kg), it effectively inhibited tumor growth; when the dose increased to 4.0 mg/kg, the tumor shrank and left small fibrosis-like tissue. Over the whole monitoring period, the body weights of the mice in all treatment groups were recorded (Figure 8B), and no significant weight loss was observed, indicating that JVM-PE24X7 treatment was well tolerated by the animals, even at a dose as high as 4.0 mg/kg. Figure 8C shows the results of the histological analysis. Unlike the control group, in the immunotoxin-treatment group, the tumor tissue morphology was damaged, but other major organs, including the heart, liver, spleen, lung and kidney, were not obviously affected. These results further indicate that JVM-PE24X7 has good tumor-killing ability and is safe for therapeutic use.

### 2.8. Off-Target Toxicity

We further examined the off-target toxicity of JVM-PE24X7. Here, to investigate the effect of replacing PE38 with PE24X7 on the off-target toxicity of the immunotoxins and analyze the effects of free PE24X7 itself, we prepared and examined the immunotoxin JVM-PE38, which fused the conventional PE toxin PE38 with the sdAb JVM (Figure S3), and the free toxin PE24X7 without JVM. We selected tumor-free BALB/c mice for this examination and injected them with JVM-PE24X7, JVM-PE38 or PE24X7 at different doses (1.5, 3.0, 10, 15 and 20 mg/kg) via the tail vein. The physiological status and death of the mice were observed over two weeks after drug administration.

As shown in Table 1, the mice injected with 3.0 mg/kg, 10 mg/kg or 15 mg/kg JVM-PE24X7 all showed a normal performance. However, when the dose increased to 20 mg/kg, some of the animals showed rough fur and apathy and died within 7 days. The maximum tolerable dose (MTD) of JVM-PE24X7 was therefore estimated to be approximately 15–20 mg/kg. When the mice were injected with 3.0 mg/kg JVM-PE38, all of them died within 7 days. The MTD of JVM-PE38 was thus estimated to be less than 3.0 mg/kg. These data show that JVM-PE38 has substantially higher off-target toxicity than JVM-PE24X7. The free PE24X7 toxin has relatively low off-target toxicity; all of the mice survived and showed normal physiological activity after injection with the free toxin PE24X7 at a dose of 20 mg/kg.

## 3. Materials and Methods

### 3.1. Reagents

3-(4,5-Dimethylthiazol-2-yl)-2,5-diphenyltetrazolium bromide (MTT), 4,6-diamidino-2-phenylindole (DAPI), 3,3′,5,5′-tetramethylbenzidine (TMB) and isopropyl-β-D-1-thiogalactopyranoside (IPTG) were obtained from Solarbio (Beijing, China). A Live & Dead Viability/Cytotoxicity Assay Kit was purchased from US Everbright Inc. (Suzhou, China). Rabbit anti-PE antibody was obtained from Sigma (cat# P2318-1ML, St. Louis, MO, USA). HRP-labeled goat anti-rabbit IgG antibody and HRP-labeled mouse anti-His antibody (cat# CW0103) were purchased from CoWin Biosciences (Jiangsu, China). Human PSMA protein was purchased from ACRO Biosystems (cat# PSA-H5264, Beijing, China). Rabbit anti-PSMA antibody (cat# A3220) and FITC-labeled goat anti-rabbit IgG (cat# AS011) were purchased from ABclonal (Wuhan, China). RPMI 1640 medium and DMEM medium were purchased from GIBCO BRL (Grand Island, NY, USA). Anti-caspase-3 Rabbit mAb (cat #14220) was purchased from Cell Signaling Technology (Danvers, MA, USA). All other materials were obtained from Tianjin Bestbay Biology Company (Tianjing, China).

### 3.2. Cell Lines and Animals

The human PCa cell lines LNCaP, C4-2B, 22Rv1 and PC-3 were purchased from the Typical Culture Preservation Commission Cell Bank of the Chinese Academy of Sciences (Shanghai, China). Here, LNCaP, C4-2B, and 22Rv1 cells were PSMA-positive, while PC-3 cells were PSMA-negative. LNCaP and C4-2B cells were cultured in RPMI 1640 medium, and 22Rv1 and PC-3 cells were cultured in DMEM. Both media were supplemented with penicillin, streptomycin and 10% heat-inactivated fetal bovine serum. The cells were maintained in culture at 37 °C with 5% CO_2_ in air and 95% humidity. A solution of 0.05% trypsin and 0.02% EDTA in PBS was used for cell detachment.

NOD-SCID mice (male, 5 to 6 weeks old) and BALB/c mice (male, 5 to 6 weeks old) were purchased from Vital River Laboratory Animal Technology Co., Ltd. (Beijing, China). All mice were housed and treated in accordance with the guidelines of the Committee on Animals of Nankai University (Tianjin, China).

### 3.3. Expression and Purification of the Anti-PSMA Immunotoxin JVM-PE24X7

An expression plasmid for the anti-PSMA immunotoxin JVM-PE24X7 was constructed using the codon-optimized nucleotide sequence of the PE24X7 nucleotide sequence [34,35] and anti-PSMA sdAb JVM [36], and their sequences were inserted into the pET22b (+) vector (Novagen, Madison, WI, USA) with NcoI and XhoI restriction enzyme sites (the sequences of JVM-PE24X7 and JVM are shown in the supporting information). The plasmid was expressed in BL21 (DE3) *E. coli* cells. Expression was carried out by shaking at 220 rpm and 16 °C for 8 h with induction by 0.5 mM IPTG. After centrifugation (4000 rpm, 20 min, 4 °C), the pellets were resuspended in PBS and crushed with a high-pressure homogenizer. The extraction was centrifuged at 18,000 rpm for 40 min, and the supernatant was purified by a nickel chelate affinity chromatography column to obtain the immunotoxin JVM-PE24X7. The purity of the protein was analyzed by SDS-PAGE. For Western blot analysis, the purified protein was detected with an HRP-labeled mouse anti-His antibody (1:1000 dilution). MALDI-TOF mass spectrometry was used to verify the molecular weight of the immunotoxin JVM-PE24X7. The anti-PSMA sdAb JVM was obtained and verified in a way similar to that of JVM-PE24X7. To determine its storage stability, PSMA-positive C4-2B cells were treated with JVM-PE24X7 stored under different storage conditions. Cell viability was determined by MTT assay. The EC_50_ under each storage condition and relative activity (EC_50_ relative to the freshly purified JVM-PE24X7) were calculated.

### 3.4. Analysis of the Cellular Expression of PSMA by CLSM and Western Blot Analysis

LNCaP, C4-2B, 22Rv1 and PC-3 cells were plated in 8-well chamber slides with 1× 10^4^ cells in medium (0.5 mL) in each well. After 24 h, the cells were washed with PBS and fixed in 4% paraformaldehyde. The cells were permeabilized with 0.2% Triton-X 100 and blocked with 1% bovine serum albumin (BSA) in PBS for 1 h. The cells were then incubated with a rabbit monoclonal anti-PSMA antibody at a dilution of 1:200 to a final concentration of 1.0 μg/mL. After washing with PBS, the cells were incubated with a goat anti-rabbit IgG conjugated with FITC in blocking solution at a final concentration of 2.0 μg/mL for 1 h at room temperature in the dark. After staining with DiI and DAPI, respectively, the slides were washed three times with PBS and visualized by confocal laser scanning microscopy (CLSM, Leica TCS SP5, Leica, Germany).

For Western blot analysis, LNCaP, C4-2B, 22Rv1 and PC-3 cells were washed with PBS and lysed with RIPA lysis and extraction buffer. Protein concentrations were determined with Bio-Rad Protein Assay Dye Reagent Concentrate, and the protein samples were subjected to 12.5% SDS-PAGE, followed by transfer to PVDF membranes. The membranes were blocked using 5% nonfat milk and incubated separately for 2 h at room temperature with primary antibodies against PSMA and β-actin. The membranes were then washed and probed with a 1:2000 dilution of HRP-conjugated secondary antibody and visualized by ECL (Millipore, Billerica, MA, USA). Image-Pro Plus software (version X; Media Cybernetics, Silver Springs, MD, USA) was used to quantify the intensity from Western blotting analysis.

### 3.5. Analysis of Binding Affinity Based on ELISA

First, the binding affinity of the prepared JVM-PE24X7 to the PSMA receptor was quantified by ELISA with the PSMA protein coated on the plate. The human PSMA protein (1 µg/well) was added to a 96-well plate and incubated overnight. After washing and incubation with blocking solution for 2 h at room temperature, the purified immunotoxin was added with a dilution gradient. For the JVM-PE24X7 test, 1 h later, rabbit anti-PE antibody (1:1000 dilution) was added, followed by incubation for another 1 h. After washing, the plate was incubated with HRP-conjugated goat anti-rabbit IgG for 1 h. For the JVM test, 1 h after the addition of blocking solution, HRP-labeled mouse anti-His antibody was added, followed by incubation for another 1 h. Before the reaction was stopped with 2.0 N H_2_SO_4_ (100 µL/well), the plates were developed with 100 µL/well TMB, and the absorbance was measured at 450 nm.

The binding affinities of JVM-PE24X7 and JVM for the PSMA receptor were also quantified by cell-based ELISA. PSMA-positive LNCaP cells (2 × 10^5^ cells/well) and PSMA-negative PC-3 cells (1 × 10^5^ cells/well) were inoculated in 96-well plates for 24 h. After washing with PBS 3 times, the cells were fixed with a paraformaldehyde solution, and then, JVM-PE24X7 or JVM was added. The plates were developed and measured as described above.

### 3.6. In Vitro Cytotoxicity Assays

The MTT assay was used to determine the PSMA-specific cytotoxicity of the immunotoxin JVM-PE24X7. Cells were seeded in 96-well plates (5000 cells/well) and cultured for 24 h at 37 °C. The cells were then exposed to a graded JVM-PE24X7 series (from 0.01 pM to 1.0 μM) for 72 h or with JVM or PBS as controls. MTT solution (5 mg/mL in PBS) was added to each well. The plates were incubated for 4 h at 37 °C. After the supernatant had been removed, the produced formazan crystals were dissolved in dimethyl sulfoxide (DMSO) for 10 min at room temperature, and the absorbance at both 490 nm and 570 nm was measured. Cells without immunotoxin treatment were used as controls, and the EC_50_ values were calculated using GraphPad Prism software. For receptor-blocking assays, cells were preincubated for 1 h with 1.0 µM unmodified anti-PSMA sdAb JVM to block the PSMA receptors prior to the addition of serial dilutions of the immunotoxin JVM-PE24X7.

An APC Annexin V/PI apoptosis detection kit (BioLegend, San Diego, CA, USA) was used to analyze the cytotoxicity of the immunotoxin. PSMA-positive C4-2B cells and PSMA-negative PC-3 cells were seeded in 12-well plates at a density of 1 × 10^5^ overnight and treated with 100 pM or 100 nM JVM-PE24X7, JVM or PE24X7 for 24, 48 or 72 h. After digestion with trypsin, the cells were collected by centrifugation at 300 g for 5 min, washed with AV binding buffer and then stained with annexin V-FITC and PI at room temperature for 15–20 min. The samples were analyzed by flow cytometry.

For Western blot analysis, C4-2B cells were seeded in 12-well plates at a density of 1 × 10^5^ overnight and treated with 100 pM JVM-PE24X7 for 12, 24, 48 or 72 h. After digestion with trypsin, the cells were collected by centrifugation at 300 g for 5 min, washed with PBS and lysed with RIPA lysis and extraction buffer. Protein concentrations were determined with Bio-Rad Protein Assay Dye Reagent Concentrate, and then the protein samples were subjected to 12.5% SDS-PAGE, followed by transfer to PVDF membranes. The membranes were blocked using 5% nonfat milk and incubated separately for 2 h at room temperature with primary antibodies against caspase-3 and β-actin. The membranes were then washed and probed with a 1:2000 dilution of HRP-conjugated secondary antibody and visualized as described before.

### 3.7. In Vitro Observation with Live/Dead Staining

Live/dead staining was used to visualize the cytotoxicity of JVM-PE24X7. Here, LNCaP, C4-2B, 22Rv1 and PC-3 cells were plated in 96-well plates (5000 cells/well) and incubated for 24 h at 37 °C. After the medium was replaced with fresh medium, the cells were incubated with JVM-PE24X7 at 37 °C under 5% CO_2_ for 72 h or with JVM or PBS as controls. Then, the plates were washed three times with PBS and stained with calcein-AM:ethidium homodimer-1 (EthD-1):PBS = 1:4:1000 for 15–20 min. The cells were visualized with an inverted microscope (DMI 4000B, Leica, Germany).

### 3.8. Analysis of the Internalization of JVM-PE24X7 into Cells

Here, JVM-PE24X7 was first labeled with the fluorescent dye rhodamine B. LNCaP and PC-3 PCa cells grown to 60 to 70% confluence on four-chamber glass slides were used for endocytosis analysis. The cells were incubated with rhodamine B-labeled JVM-PE24X7 (0.5 μM) in complete medium for different durations (from 10 min to 180 min) or incubated with graded concentrations of rhodamine B-labeled JVM-PE24X7 (from 0 μM to 0.5 μM) for 180 min. After removal of the media, the cells were washed three times with PBS, fixed with 10% formalin for 10 min, stained with DAPI for 5 min, and rinsed three times with PBS. The cells were then observed by CLSM. For quantitative analysis of the internalization of JVM-PE24X7, JVM-PE24X7 was first labeled with FITC and then incubated (0.5 μM) with LNCaP and PC-3 PCa cells in complete medium for different durations (from 10 min to 180 min) or with graded concentrations (from 0 μM to 0.5 μM) for 180 min. CellQuest Pro (BD Biosciences, San Jose, CA, USA) was used for data acquisition, and FlowJo (Tree Star Inc., Ashland, OR, USA) was used for data analysis.

### 3.9. In Vivo Antitumor Activity of JVM-PE24X7

Subcutaneous C4-2B xenograft tumors with high expression levels of the PSMA receptor were used to evaluate the in vivo antitumor activity of the immunotoxin JVM-PE24X7. The model was constructed by the subcutaneous inoculation of 2 × 10^7^ C4-2B cells per mouse in 100 μL of PBS mixed with 100 μL of Matrigel (Collaborative Biomedical Products, Chicago, IL) into the right flank of male NOD-SCID mice. When the tumor volumes reached approximately 140 mm^3^, the mice were divided randomly into 5 groups (*n* = 5). In the four treatment groups, the mice were administered 0.5, 1.0, 2.0 or 4.0 mg/kg JVM-PE24X7 via the tail vein every three days for a total of five doses on days 1, 4, 7, 10 and 13. In the control group, the mice were given PBS at the same time points. Tumor growth was measured with a digital caliper, and tumor volumes were calculated using the following formula: length × (width)^2^/2. The body weights of the animals were also recorded throughout the whole experiment.

At the end of treatment period, on the 37th day, the mice in the group treated with 4.0 mg/kg JVM-PE24X7 and control group were euthanized. Their tumor tissues and major organs were collected and fixed with 4% paraformaldehyde. The samples were used to prepare paraffin sections that were then stained with hematoxylin and eosin (H&E), and histopathological analysis was performed with a Nikon Eclipse E100 microscope (Tokyo, Japan).

### 3.10. Off-Target Toxicity Analysis

BALB/c mice (*n* = 5) were intravenously injected with a single dose of JVM-PE24X7, JVM-PE38 or PE24X7 (1.5 mg/kg, 3.0 mg/kg, 10 mg/kg, 15 mg/kg or 20 mg/kg). The physiological changes and death of the mice were continuously observed over two weeks after injection.

### 3.11. Statistical Analysis

All data are presented with the standard error of the mean (SEM), and statistical analysis was performed using Student’s *t*-test. Differences with a value of *p* less than 0.05 were considered statistically significant.

## 4. Conclusions

In this study, we designed and prepared a new anti-PSMA immunotoxin molecule based on the sdAb JVM and the improved PE toxin PE24X7 and evaluated the potential of this molecule for PCa treatment through detailed cell- and animal-based experiments. By using a sdAb as the target ligand, the designed immunotoxin, JVM-PE24X7, was expressed well in the *E. coli* system in its soluble form, and the product was very stable; this process avoided the complex renaturation procedure required in the conventional strategy for immunotoxin preparation. JVM-PE24X7 showed good binding ability towards the PSMA receptor and could very efficiently and selectively kill PSMA-positive tumor cells at an extremely low concentration, with an EC_50_ value of 15.3 pM against receptor-positive LNCaP cells, showing more than 300-fold cytotoxic selectivity between receptor-positive and receptor-negative cells. Moreover, because the improved PE toxin PE24X7, which has substantially reduced off-target toxicity and immunogenicity, was adopted as the effector moiety, prepared JVM-PE24X7 has a wide therapeutic window and completely inhibited PCa tumor growth in an animal model at a low dose (5 × 0.5 mg/kg; once every three days) with an MTD that was greater than 15 mg/kg. These results show that JVM-PE24X7 is of great value for PCa treatment. Moreover, this study also shows that the design of immunotoxin molecules based on sdAbs and the new PE24X7 toxin is very convenient and can significantly improve the therapeutic window for immunotoxins.

## Figures and Tables

**Table 1:** Off-target toxicity of JVM-PE24X7, JVM-PE38 and PE24X7.

Dead/Total Mice					
Injected Dose (mg/kg)	1.5	3.0	10	15	20
JVM-PE24X7	—	0/5	0/5	0/5	3/5
JVM-PE38	0/5	5/5	—	—	—
PE24X7	—	—	—	0/5	0/5

## Data Availability

Not applicable.

## Supplementary Materials

The following are available online at https://www.mdpi.com/article/10.3390/ijms22115501/s1.
