# Supplementary material for: A Single-Domain Antibody-Based Anti-PSMA Recombinant Immunotoxin Exhibits Specificity and Efficacy for Prostate Cancer Therapy
Source: Int J Mol Sci. 2021 May 23;22(11):5501. doi: 10.3390/ijms22115501 (PMC8197099; doi:10.3390/ijms22115501)
Supplement: Supplementary file 1 [file ijms-22-05501-s001.zip › ijms-1208868-supplementary.pdf]

Supporting Information

A Single-Domain Antibody-Based Anti-PSMA Recombinant Immunotoxin Exhibits Specificity and Efficacy for Prostate Cancer Therapy

Yutong Xing, Keyuan Xu, Shixiong Li, Li Cao, Yue Nan, Qiyu Li, Wenjing Li and Zhangyong Hong*

State Key Laboratory of Medicinal Chemical Biology, College of Life Sciences, Nankai University, Tianjin 300071, China; xingyutong@mail.nankai.edu.cn (Y.X.); 1120190518@mail.nankai.edu.cn (K.X.); 2120191089@mail.nankai.edu.cn (S.L.); 1120150370@mail.nankai.edu.cn (L.C.); 2120201117@mail.nankai.edu.cn (Y.N.); qiyuli@mail.nankai.edu.cn (Q.L.); 1120160385@mail.nankai.edu.cn (W.L.).

* Correspondence: hongzy@nankai.edu.cn (Z.H.); +86-022-2349-8707 (Z.H.)

Amino acid sequence of JVM-PE24X7 and JVM-PE38 (Sequence of JVM is labeled as red and sequence of PE24X7 and PE38 is labeled as blue).

JVM-PE24X7:

HHHHHHEVQLVESGGGLVQPGGSLTLSCAASRFMISEYSMHWVRQAPGK
GLEWVSTINPAGTTDYAESVKGRFTISRDNAKNTLYLQMNSLKPEDTAVYY
CDGYGYRGQGTQVTVSSGGGGSGGGGSRHRQPRGWEQLGGSPTGAEFLG
DGGDVSFSTRGTQNWTVERLLQAHAQLEERGYVFVGYHGTFLEAAQSIVF
GGVRAASQDLAAIWAGFYIAGDPALAYGYAQDQEPDAAGRIRNGALLRV
YVPASSLPGFYRTSLTLAAPEAAGEVERLIGHPLPLALDAITGPEEEGGRLET
ILGWPLAERTVVIPSAIPTDPRNVGGDLDPSSIPDKEQAISALPDYASQPGKP
PKDEL

JVM-PE38:

HHHHHHEVQLVESGGGLVQPGGSLTLSCAASRFMISEYSMHWVRQAPGK
GLEWVSTINPAGTTDYAESVKGRFTISRDNAKNTLYLQMNSLKPEDTAVYY
CDGYGYRGQGTQVTVSSGGGGSGGGGSPEGGSLAALTAHQACHLPLETFT
RHRQPRGWEQLEQCGYPVQRLVALYLAARLSWNQVDQVIRNALASPGSG
GDLGEAIREQPEQARLALTLAAAESERFVRQGTGNDEAGAANGPADSGD
ALLERNYPTGAEFLGDGGDVSFSTRGTQNWTVERLLQAHRQLEERGYVFV
GYHGTFLEAAQSIVFGGVSARSQDLDAIWRGFYIAGDPALAYGYAQDQEP
DARGRIRNGALLRVYVPRSSLPGFYRTSLTLAAPEAAGEVERLIGHPLPLRL
DAITGPEEEGGRLETILGWPLAERTVVIPSAIPTDPRNVGGDLDPSSIPDKEQ
AISALPDYASQPGKPPKDEL

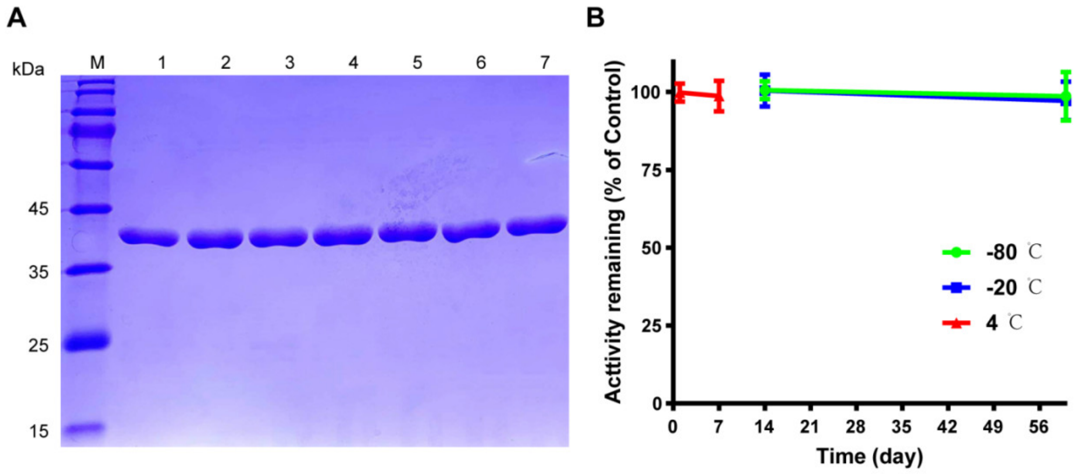

Figure S1. Storage stability analysis of JVM-PE24X7 by SDS-PAGE and cytotoxicity assay. (**A**) Freshly purified JVM-PE24X7 (lane 1) was stored at 4 °C for 24 h (lane 2) and 1 week (lane 3), at -20 °C for 2 weeks (lane 4) and 2 months (lane 5), and at -80 °C for 2 weeks (lane 6) and 2 months (lane 7). The proteins were loaded onto a 12.5% polyacrylamide gel with protein weight standards and detected by Coomassie brilliant blue staining (**B**) Evaluation of residual activity of JVM-PE24X7 after storage at different temperatures for different durations. Data is presented as a percentage of the remaining activity compared to the freshly purified JVM-PE24X7.

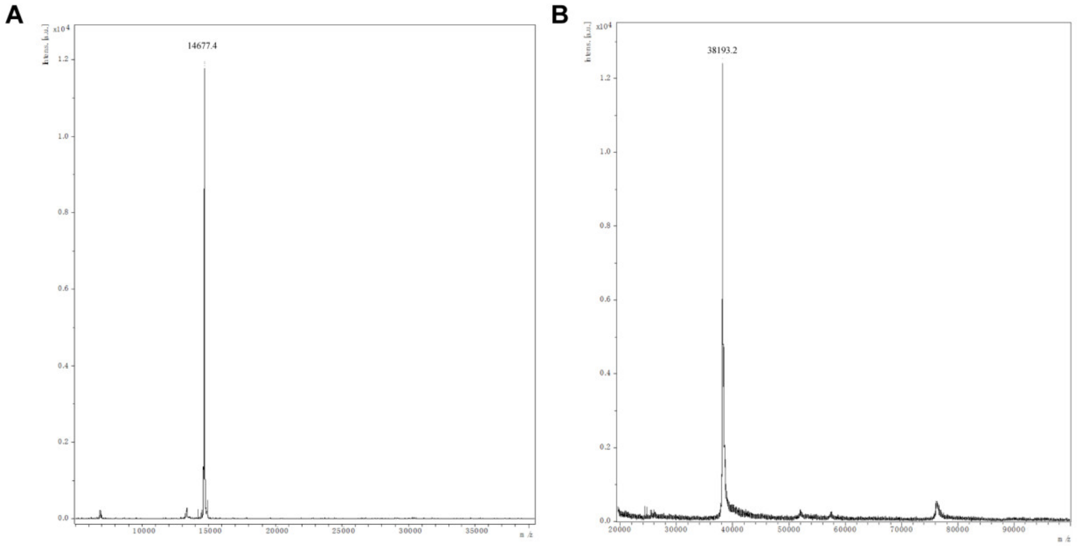

Figure S2. MALDI-TOF MS of purified JVM-PE24X7 and JVM. (A) Theoretical molecular mass of JVM-PE24X7 is 38172.4 Da and detected molecular mass is 38193.2 Da. (B) Theoretical molecular mass of JVM is 14621.4 Da and detected molecular mass is 14677.4 Da.

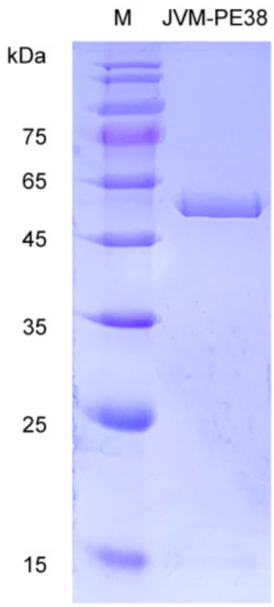

Figure S3. SDS-PAGE of purified JVM-PE38. The protein was loaded onto a 12.5% polyacrylamide gel with protein weight standards, and detected through Coomassie brilliant blue staining.

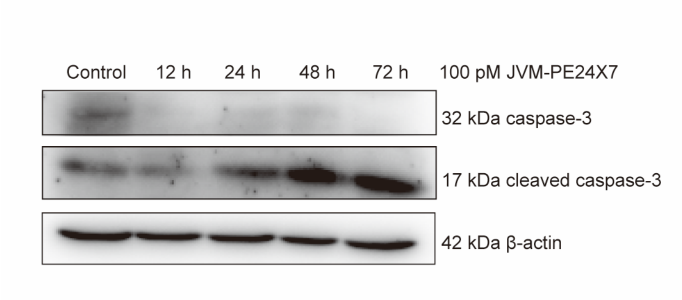

Figure S4. Western blot analyses of apoptosis-related proteins in C4-2B cells. Western blot showed the dynamic changes of caspase-3 and cleaved caspase-3 in C4-2B cells treated with 100 pM JVM-PE24X7 for 12 h, 24 h, 48 h or 72 h.
